# Prognostic Value of Baseline Neutrophil-to-Lymphocyte Ratio in Advanced Breast Cancer Patients Treated with Trastuzumab Deruxtecan: A Real-World Study

**DOI:** 10.3390/jcm15218437

**Published:** 2026-10-29

**Authors:** Zhihao He, Yifei Chen, Guohong Song, Lijun Di, Xiaoning Wu, Jichun Song, Wei Zhang, Baichuan Cao, Qin Li, Bin Shao

**Affiliations:** 1 Department of Oncology, Beijing Friendship Hospital, Capital Medical University, Beijing 100050, China; 2 Department of Breast Oncology, Key Laboratory of Carcinogenesis and Translational Research (Ministry of Education/Beijing), Peking University Cancer Hospital & Institute, No. 52 Fuchen Road, Haidian District, Beijing 100142, China

**Keywords:** HER2-positive advanced breast cancer, trastuzumab deruxtecan, antibody–drug conjugate, neutrophil-to-lymphocyte ratio, lymphocyte count, prognostic biomarker, real-world study

## Abstract

**Background:** Trastuzumab deruxtecan (T-DXd) has redefined the treatment of HER2-positive advanced breast cancer (HER2+ ABC), yet responses remain heterogeneous and accessible biomarkers are lacking. The neutrophil-to-lymphocyte ratio (NLR) is an inexpensive, routinely available systemic inflammatory marker with established prognostic value in HER2-positive disease treated with anti-HER2 monoclonal antibodies; its prognostic significance in patients receiving the antibody–drug conjugate T-DXd remains undetermined. **Methods:** This two-centre real-world study screened 146 consecutive patients with HER2+ ABC who received at least one cycle of T-DXd between January 2023 and June 2025 (data cutoff 16 October 2025) and enrolled 92 who met all eligibility criteria. The prespecified eligibility rule requiring a baseline complete blood count within 7 days before the first T-DXd dose was applied uniformly to every screened candidate; 34 candidates failed this rule (count drawn after the first dose, *n* = 16; drawn more than 7 days before, *n* = 17; collection date not recorded, *n* = 1) and one candidate was excluded because a baseline lymphocyte count at the assay lower limit rendered the derived ratio unreliable. The primary analysis evaluated baseline NLR as a continuous variable in Cox proportional-hazards regression for progression-free survival (PFS). Prespecified secondary analyses comprised median-split Kaplan–Meier analysis, quartile analysis with trend testing, individual blood components, and multivariable models. Prespecified sensitivity analyses, proportional-hazards testing, restricted cubic spline assessment of linearity, nested-model likelihood-ratio testing of the incremental value of NLR over the absolute lymphocyte count, and a centre-stratified analysis were performed. Exploratory subgroup analyses included interaction testing with false-discovery-rate correction. **Results:** Of 146 screened patients, 92 were enrolled (54 excluded); 39 PFS events occurred. Median follow-up by reverse Kaplan–Meier was 7.6 months; overall median PFS was 9.82 months, with 6- and 12-month PFS rates of 73.7% (95% CI 62.3–82.1) and 37.1% (22.6–51.6). Each one-unit increase in baseline NLR was associated with a 37% higher hazard of progression or death (HR 1.37, 95% CI 1.12–1.69, *p* = 0.0028; C-index 0.672). At the cohort median cut-off (2.157), high NLR conferred shorter PFS than low NLR (median 7.66 vs. 11.50 months; HR 2.30, 95% CI 1.21–4.38; log-rank *p* = 0.0096). Quartile analysis showed a significant dose–response trend (HR per quartile 1.58, 95% CI 1.16–2.13, *p* for trend = 0.0033), with Q4 versus Q1 HR 4.14 (1.52–11.26, *p* = 0.0053). Among individual components, only the lymphocyte count showed a significant median-split association (log-rank *p* = 0.011), and NLR provided prognostic information beyond the lymphocyte count (likelihood-ratio *p* = 0.039) and beyond a clinically driven multivariable model (likelihood-ratio *p* = 0.014; ΔC-index +0.077). In the clinically driven multivariable model (*n* = 92, 7.8 events per variable), NLR remained an independent prognostic factor (HR 1.36, 95% CI 1.09–1.70, *p* = 0.0062), and estimates were consistent across a minimal model, two extended models and a ridge-penalised model. The association was consistent across both centres (interaction *p* = 0.73). Model assumptions were satisfied (Schoenfeld *p* = 0.75; non-linearity *p* = 0.46). No subgroup interaction remained significant after false-discovery-rate correction across 17 tests. **Conclusions:** Elevated baseline NLR is an independent adverse prognostic marker for PFS among HER2+ ABC patients treated with T-DXd, and carries prognostic information beyond its individual blood components and beyond standard clinical variables. Because no comparator arm exists, NLR should be interpreted as a prognostic—not predictive—biomarker in this setting; prospective validation is required before any clinical application.

## 1. Introduction

Breast cancer remains the most frequently diagnosed malignancy and the leading cause of cancer death among women worldwide [1]. HER2-positive disease accounts for 15–20% of cases and is characterised by aggressive biology and a propensity for early recurrence and visceral and central nervous system metastasis [2]. The antibody–drug conjugate (ADC) trastuzumab deruxtecan (T-DXd; Enhertu^®^, Daiichi Sankyo Co., Ltd., Tokyo, Japan), a HER2-targeting antibody linked to a topoisomerase I inhibitor payload, has redefined the treatment landscape: in DESTINY-Breast03 it significantly outperformed trastuzumab emtansine in previously treated HER2-positive metastatic disease [3,4], in DESTINY-Breast04 it established efficacy in HER2-low disease [5], and randomised-trial meta-analyses confirm a consistent benefit across HER2-expressing advanced breast cancer [6].

Despite this efficacy, responses to T-DXd are heterogeneous: a substantial proportion of patients progress within months of initiation, and interindividual differences in progression-free survival (PFS) are wide. Accessible, inexpensive biomarkers that refine risk stratification and inform monitoring intensity are therefore of considerable clinical interest.

Systemic inflammation is a recognised determinant of tumour progression, metastasis and treatment resistance [7,8]. The neutrophil-to-lymphocyte ratio (NLR), computed from a routine complete blood count, integrates two opposing host responses: neutrophil-dominated innate inflammation, which can support tumour growth through N2-polarised pro-tumourigenic neutrophils and suppression of adaptive immunity, and lymphocyte-mediated cytotoxic surveillance, whose depletion reflects impaired anti-tumour immunity [7,8,9,10]. NLR has been validated as a prognostic marker across solid tumours in systematic reviews, meta-analyses, umbrella reviews and narrative syntheses [9,10,11,12,13,14,15], including breast cancer [10,11,16,17,18,19], and specifically in HER2-positive disease treated with anti-HER2 therapy: exploratory analyses of the CLEOPATRA trial identified baseline NLR as prognostic in trastuzumab-treated HER2-positive metastatic breast cancer [20], and independent cohorts treated with trastuzumab-based regimens reported consistent associations [21,22]. Peripheral NLR correlates with tumour-infiltrating lymphocytes and tumour-associated macrophages [23], and related composite indices such as the systemic immune-inflammation index show prognostic associations in breast cancer [24]. In non-HER2-directed settings, NLR and the absolute lymphocyte count likewise retain prognostic relevance in advanced breast cancer [25]. The evidence is not uniformly positive, however: a detailed deconstruction of NLR in early breast cancer reported a lack of prognostic utility and inconsistent biological correlates across subtypes [26], indicating that the prognostic value of NLR is context-dependent and must be established empirically in each treatment setting.

Whether the prognostic signal of baseline NLR extends to patients treated with T-DXd is unknown, and the question is not trivial. ADC efficacy depends on target-mediated internalisation and intracellular payload release, mechanisms that differ from the antibody-dependent cellular cytotoxicity and Fc-mediated immune effector functions through which host immune competence may partly modify monoclonal antibody activity. Real-world data addressing this question are scarce: contemporary real-world T-DXd cohorts have characterised clinical outcomes, drug–drug interactions and the influence of body composition on toxicity and response [27,28], but none has evaluated baseline inflammatory indices with prespecified analyses and formal model diagnostics.

We therefore conducted a two-centre real-world study of baseline NLR in HER2+ ABC patients treated with T-DXd, designed with five features intended to make the result interpretable and reproducible: (i) NLR was evaluated primarily as a continuous variable to avoid dichotomisation-related information loss; (ii) the primary endpoint, secondary analyses, sensitivity analyses and model diagnostics were prespecified before analysis, while the covariate set of the primary multivariable model was established on clinical grounds during revision in response to peer review and is described accordingly as a clinically driven model rather than as prespecified; (iii) the incremental prognostic value of NLR over its own mathematical constituent, the absolute lymphocyte count, was quantified by nested-model comparison rather than inferred from significance levels across separate models; (iv) the eligibility rule governing the timing of the baseline blood count was applied uniformly to every screened candidate and every exclusion is reported with its count and reason; and (v) all reported estimates were re-derived de novo from the source dataset, using a single analysis set consistently throughout.

## 2. Materials and Methods

### 2.1. Study Design and Patient Population

This retrospective two-centre cohort study was conducted at Peking University Cancer Hospital and Beijing Friendship Hospital. Consecutive patients who received at least one cycle of T-DXd for advanced or metastatic HER2-positive breast cancer between 14 January 2023 and 25 June 2025 were screened; data were censored at 16 October 2025.

Inclusion criteria were: (i) histopathologically confirmed invasive breast cancer with HER2 positivity according to ASCO/CAP guidelines (immunohistochemistry 3+, or 2+ with confirmed amplification by in situ hybridisation) [29,30]; (ii) at least one cycle of T-DXd for advanced or metastatic disease; (iii) a baseline complete blood count obtained within 7 days before the first T-DXd administration; and (iv) at least one post-baseline tumour assessment permitting evaluation of PFS.

For clarity, the following operational definitions were applied. Advanced disease denotes locally advanced, inoperable disease, including extensive regional nodal involvement or chest-wall or skin involvement not amenable to curative-intent surgery or radiotherapy. Metastatic disease denotes documented spread to solid organs (liver, lung, brain), to bone, or to non-regional lymph nodes.

Exclusion criteria were: (i) incomplete baseline clinical or laboratory data, defined as the absence of any variable required for the primary analysis—the baseline complete blood count or the NLR derived from it; (ii) follow-up shorter than one month with no post-baseline assessment, or an unascertainable progression status or last-assessment date—patients who progressed or died within the first month were retained and counted as events at their recorded time-to-event; (iii) failure of the prespecified timing rule, that is, a baseline complete blood count drawn after the first T-DXd dose, drawn more than 7 days before it, or with an unrecorded collection date—this rule was applied uniformly to all screened candidates, including those whose counts were otherwise complete; and (iv) a baseline laboratory value outside the physiologically plausible range such that the derived ratio is unreliable, as specified in Section 2.3.

**Figure 1:**
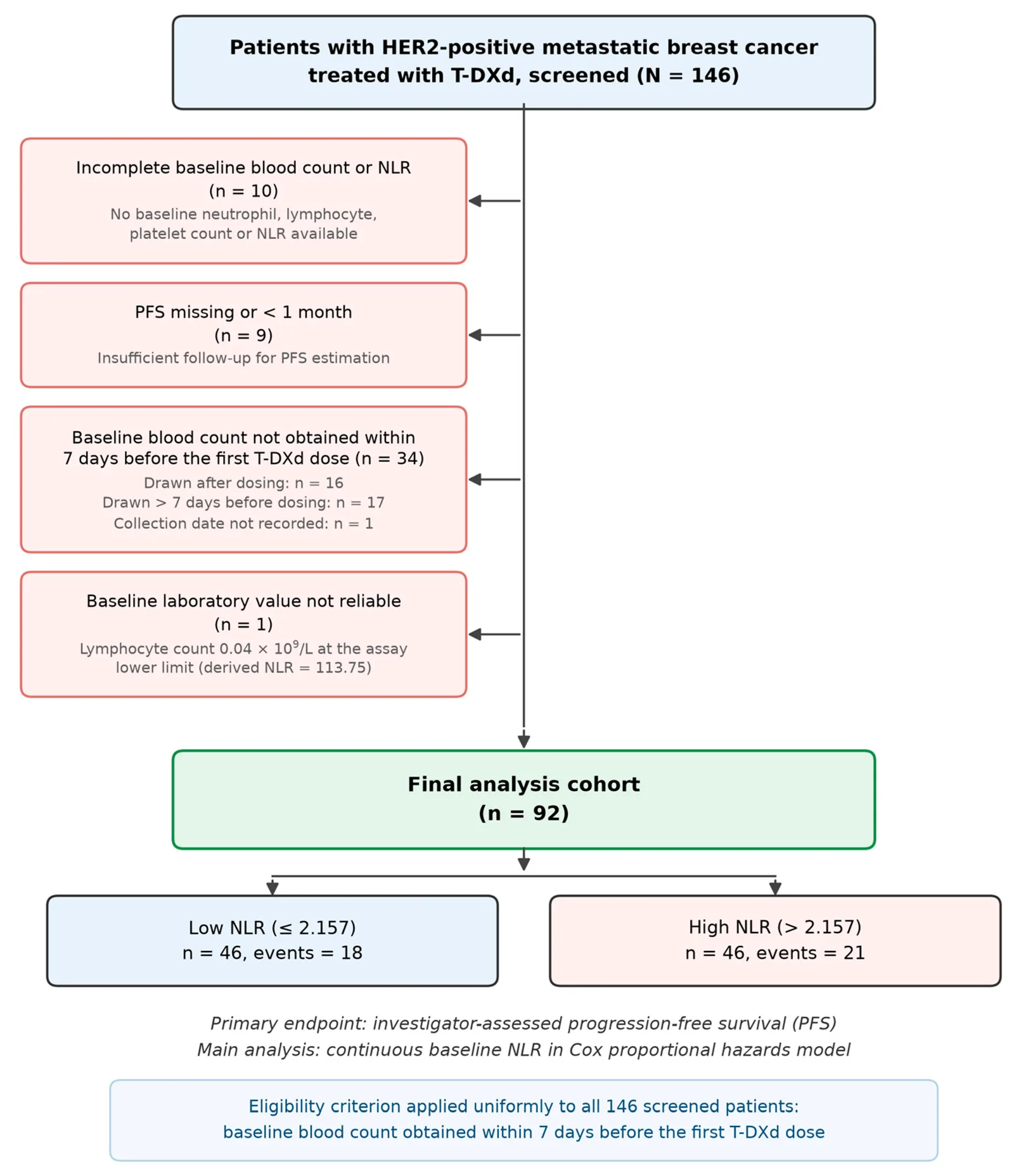
Participant flow diagram. The eligibility rule requiring a baseline complete blood count within 7 days before the first T-DXd dose was applied uniformly to all 146 screened patients; exclusions at each step are shown with counts and reasons.

Of 146 screened patients, 54 were excluded (incomplete baseline blood counts or baseline NLR unavailable, *n* = 10; follow-up shorter than one month or PFS missing, *n* = 9; baseline blood count not obtained within 7 days before the first T-DXd dose, *n* = 34, comprising counts drawn after the first dose in 16, drawn more than 7 days before it in 17, and an unrecorded collection date in 1; and an unreliable baseline laboratory value, *n* = 1), leaving 92 patients in the analysis cohort (Figure 1). All 92 had complete baseline NLR, PFS and event status, and all were included in the primary analysis; no patient was excluded after enrolment.

The study was approved by the Institutional Review Board of Peking University Cancer Hospital (approval No. 2025YJZ20, approved 14 April 2025), at which the great majority of the cohort was treated and from which the source data were drawn; retrospective data extraction began on 1 July 2025, after that approval had been granted, and was complete by the data cutoff of 16 October 2025. The Institutional Review Board of Beijing Friendship Hospital subsequently approved the study (approval No. 2026-P2-263-01, reviewed 17 May 2026 and approved 18 May 2026) for the analysis and publication phase covering the patients treated at that centre. The requirement for written informed consent was waived by both boards because the study involved no intervention and used de-identified data collected during routine clinical care. The study is reported according to the STROBE statement.

### 2.2. Data Collection and Variable Definitions

Demographic, clinicopathological, treatment and laboratory data were extracted from electronic medical records. Hormone receptor (HR) positivity was defined as at least 1% nuclear staining for oestrogen or progesterone receptor. The site-specific metastasis variables (liver, lung and brain) were derived from the metastatic sites documented before the first T-DXd administration and were verified against the corresponding source text field in every patient (92/92 concordant for each of the three sites). The visceral metastasis variable is the categorical field as recorded in the source dataset; it was not re-derived from the three site-specific variables, and the two are therefore not identical (81 patients flagged visceral-positive versus 77 with any of liver, lung or brain involvement). This discrepancy is reported explicitly in the footnote to Table 1 and in the Limitations. The number of prior systemic treatment lines, prior anti-HER2 treatment lines, and prior exposure to trastuzumab, pertuzumab, tyrosine kinase inhibitors and T-DM1 were recorded. Body mass index (BMI) was computed from the height and weight recorded in the source study record and dichotomised at 25 kg/m^2^ [31]; the analysis dataset used for modelling contained no height or weight values, so BMI was taken from the source record and was evaluable only in the 84 patients with those values recorded. ECOG performance status was recorded on the 0–3 scale and dichotomised at ≥1 for analysis.

Disease-free survival (DFS) was defined as the interval from initial curative-intent treatment (surgery and/or completion of primary systemic therapy) to the date of first documented locoregional or distant recurrence. DFS is applicable only to patients who received curative-intent treatment and subsequently relapsed; it is undefined for patients presenting with de novo metastatic disease (*n* = 28), who were therefore excluded from DFS-based analyses, together with 7 patients for whom the recurrence date was not recorded, leaving 57 patients with evaluable DFS. DFS was dichotomised at 2 years, with DFS < 2 years as the reference category.

### 2.3. Blood Sample Collection, Laboratory Parameters and Data-Quality Rules

Peripheral venous blood was collected under routine standardised conditions within 7 days before the first T-DXd administration. Where multiple complete blood counts fell within this window, the measurement closest to the date of T-DXd initiation was selected. Absolute neutrophil, lymphocyte and platelet counts (×10^9^/L) were obtained from standardised automated haematology analysers. NLR was calculated as neutrophils divided by lymphocytes and the platelet-to-lymphocyte ratio (PLR) as platelets divided by lymphocytes; computed values were verified against the recorded values.

Two data-quality rules were applied at the screening stage, uniformly to all candidates. First, the timing rule of inclusion criterion (iii): for every screened candidate, the interval between the collection date of the designated baseline complete blood count and the date of the first T-DXd dose was computed from the source records, and candidates outside the interval [−7, 0] days were excluded under criterion (iii); the counts of each failure mode are reported in Section 2.1 and Figure 1. Second, a plausibility rule: candidates whose baseline counts lay outside physiologically plausible ranges (for example, a lymphocyte count at the assay lower limit, implying a derived ratio of implausible magnitude) were treated as having an unreliable baseline laboratory value and were excluded under criterion (iv). One screened candidate met this rule (lymphocyte count 0.04 × 10^9^/L, derived NLR 113.75, more than an order of magnitude above the cohort maximum of 6.79); the influence of this single value was examined in a prespecified sensitivity analysis (Section 3.9).

Information on concurrent or recent corticosteroid or G-CSF administration, active infection or other concurrent inflammatory conditions at the time of sampling, and the exact interval since the last prior systemic therapy was not systematically captured; this is addressed as a principal limitation.

### 2.4. Treatment

T-DXd was administered intravenously at 5.4 mg/kg every 3 weeks until disease progression or unacceptable toxicity, with dose reduction, interruption or discontinuation according to the prescribing information and investigator judgement; where reduction was required, it followed the approved stepwise schedule (5.4 → 4.4 → 3.2 mg/kg). Per-patient dose-intensity records were not systematically captured and are therefore not reported.

### 2.5. Endpoints

The primary endpoint was PFS, defined as the time from T-DXd initiation to radiographic or clinical progression assessed per RECIST version 1.1 [32], or to death from any cause, whichever occurred first; patients without an event were censored at the date of last assessment. Because death ascertainment was incomplete in this retrospective cohort (two deaths recorded, of which one coincided with recorded progression), PFS was in practice driven by progression events; a composite endpoint using all recorded deaths was therefore evaluated as a prespecified sensitivity analysis. Prespecified secondary endpoints were PFS at 3, 6, 9, 12, 18 and 24 months with 95% confidence intervals and numbers at risk, and the association of PFS with individual blood components and with NLR categorised at the cohort median and at quartiles.

### 2.6. Statistical Analysis

The analytical plan was prespecified with respect to the exposure, the endpoint and the family of analyses: NLR was evaluated primarily as a continuous variable in Cox proportional-hazards regression to avoid the information loss and threshold instability inherent in dichotomisation. As a prespecified secondary stratification, NLR was categorised at the median of the analysis cohort (2.157, yielding groups of 46 and 46); because PFS is a censored time-to-event outcome and no external or training cohort was available, no receiver-operating-characteristic-derived cut-off was attempted, and the median-based stratification is reported transparently as a reproducible, internally consistent categorisation. Quartile analysis reported the hazard ratio for each quartile relative to the first, together with a trend test treating quartile rank as an ordinal variable.

Kaplan–Meier estimates were compared with log-rank tests. Landmark PFS rates were reported with 95% confidence intervals computed on the complementary log–log scale, together with the number of patients at risk and the cumulative number of events at each landmark; median follow-up was estimated by the reverse Kaplan–Meier method.

Univariable Cox models were fitted for all candidate covariates. The primary multivariable model is a clinically driven model established during revision in response to peer review, not a model whose exact covariate set was fixed before any outcome analysis; it is therefore described throughout as the clinically driven multivariable model rather than as prespecified. On clinical grounds, it incorporates performance status, the source-record visceral-metastasis field, hormone receptor status and age (dichotomised at >60 years) together with NLR; disease-free survival was deliberately omitted from the primary model because it is evaluable in only 57 of 92 patients, and a model retaining DFS is reported as a complete-case sensitivity analysis. A minimal model (NLR, performance status, visceral metastasis), two extended models (adding liver and brain involvement together with either prior anti-HER2 lines or total treatment lines) and a ridge-penalised Cox model with the clinically driven covariate set are reported alongside. Events per variable are reported for every model.

Handling of missing data. No imputation was performed. Every binary covariate used in any analysis was re-derived directly from its source variable at the time of analysis, and patients for whom the source variable was missing or undefined were excluded from the analyses containing that covariate (complete-case analysis on a per-covariate basis); the number contributing to each analysis is reported in every table. Specifically, DFS was evaluable in 57 patients (undefined in the 28 with de novo metastatic presentation and unrecorded in 7 others), prior anti-HER2 treatment lines in 73, Ki-67 in 83, body-mass index in 84 (computed from height and weight recorded in the source record) and total treatment lines in 84; all remaining covariates and the primary exposure were complete in all 92 patients. We note that an earlier draft of this work had used pre-computed binary indicator fields from the source dataset in which missing source values had been recoded as belonging to one of the two categories; this error was identified during revision, all affected variables were re-derived from their source values as described above, and all affected estimates were recomputed.

Model assumptions were tested formally. The proportional-hazards assumption was assessed for NLR and for each covariate in the final model using Schoenfeld residuals with rank, Kaplan–Meier and identity time transforms. The linearity of the association between NLR and the log hazard was assessed by restricted cubic splines with three, four and five knots placed at percentiles of the observed distribution, compared with the linear specification by likelihood-ratio test and by a quadratic term; the estimated hazard ratio across the observed NLR range is presented with pointwise 95% confidence bands.

Because the lymphocyte count is a mathematical constituent of NLR, the incremental prognostic value of NLR was quantified by nested-model comparison rather than by comparing significance levels across separate univariable models: likelihood-ratio tests, Akaike information criterion (AIC) and Harrell’s C-index were computed for models containing the lymphocyte count alone, NLR alone, both, and—for completeness—the neutrophil count alone and with NLR, in both directions, and for the clinically driven model with and without NLR.

Subgroup analyses were exploratory across hormone receptor status, visceral metastasis (both the source-record field and the reconstructed liver/lung/brain definition), liver, lung and brain metastasis, body-mass index, age, performance status, DFS category, prior anti-HER2 treatment lines, total treatment lines, metastatic site number, Ki-67, de novo stage IV status and study centre; each stratum was restricted to patients with the stratifying variable recorded, and the denominator for every stratum is reported in Table 2. For each stratifying variable, an exposure-by-subgroup interaction term was tested; because seventeen interaction tests were performed, *p*-values were additionally adjusted by the Benjamini–Hochberg false-discovery-rate procedure, and both raw and adjusted values are reported. Subgroup-specific *p*-values are not interpreted as evidence of effect heterogeneity; effect estimates with 95% confidence intervals are presented as the primary summary.

The centre was identified from the format of the anonymised patient identifier: prefixed identifiers correspond to Peking University Cancer Hospital (*n* = 74) and numeric identifiers to Beijing Friendship Hospital (*n* = 18), consistent with the identifier-numbering convention of each institution’s medical-record system. A centre-stratified analysis was performed: the centre effect on PFS, the NLR–PFS association adjusted for centre, and an NLR-by-centre interaction term were estimated, and subgroup-specific estimates are reported for each centre.

All tests were two-tailed; *p* < 0.05 was considered statistically significant. Analyses were performed in Python (version 3.14.6) using the lifelines package (version 0.30.3); data handling and figure generation additionally used pandas (version 2.3.3), NumPy (version 2.5.2), SciPy (version 1.18.0) and Matplotlib (version 3.11.1). All analyses were derived de novo from the source dataset.

## 3. Results

### 3.1. Study Population and Baseline Characteristics

The participant flow is shown in Figure 1. Of 146 screened patients, 92 met all eligibility criteria and formed the analysis cohort; 39 PFS events and 53 censored observations contributed to the primary analysis. Median follow-up by the reverse Kaplan–Meier method was 7.6 months (interquartile range 4.3–8.5 among censored patients). No patient in the analysis cohort had PFS shorter than one month (minimum 1.02 months).

Baseline characteristics are summarised in Table 1. Median age was 60.5 years (range 34–78); 63.0% were HR-positive; 88.0% were flagged as having visceral metastasis in the source dataset (83.7% had liver, lung or brain involvement; see the Table 1 footnote), with liver, lung and brain involvement in 46.7%, 42.4% and 42.4% respectively; 30.4% presented with de novo stage IV disease. The median number of prior anti-HER2 treatment lines was 3 (IQR 3–4, range 2–14), and prior exposure to trastuzumab, pertuzumab, tyrosine kinase inhibitors and T-DM1 was documented in 94.9%, 83.3%, 82.3% and 35.6% of patients with available data. Median baseline NLR was 2.16 (IQR 1.52–2.72, range 0.63–6.79). Seventy-four patients (80.4%) were treated at Peking University Cancer Hospital and 18 (19.6%) at Beijing Friendship Hospital.

### 3.2. Follow-Up and Overall Progression-Free Survival

Overall median PFS was 9.82 months. Landmark PFS rates were 89.8% (95% CI 81.3–94.6) at 3 months with 74 patients at risk, 73.7% (62.3–82.1) at 6 months with 46 at risk, 55.7% (42.1–67.3) at 9 months with 22 at risk, 37.1% (22.6–51.6) at 12 months with 10 at risk, and 24.7% (11.5–40.6) at 18 months with 4 at risk (Table 3). Two deaths were recorded during follow-up; because death ascertainment was incomplete, overall survival was not analysed, and the composite endpoint was evaluated only as a sensitivity analysis (Section 3.9).

### 3.3. Primary Endpoint: Baseline NLR as a Continuous Variable

**Figure 2:**
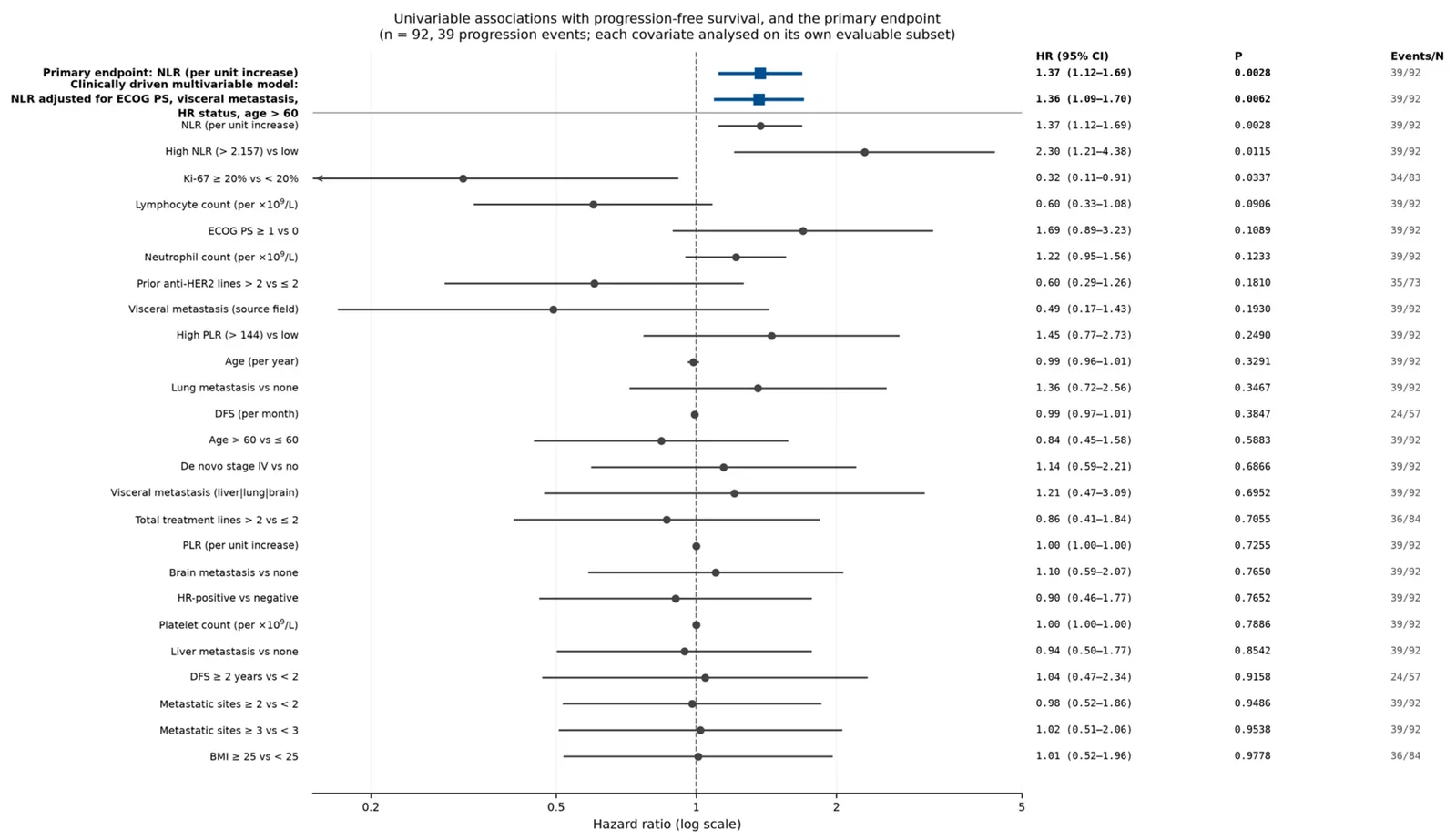
Forest plot of hazard ratios for progression-free survival. Rows above the horizontal rule: the primary endpoint (baseline NLR as a continuous variable) and the NLR term of the clinically driven multivariable model (square markers). Rows below the rule: univariable analyses of candidate covariates, each fitted on the patients evaluable for that covariate (circle markers). Horizontal lines denote 95% confidence intervals; where a confidence interval exceeds the axis limits it is truncated and indicated by an arrow. The dashed vertical line is HR = 1.0. Blood-component axes are in units of ×10^9^/L.

Each one-unit increase in baseline NLR was associated with a 37% higher hazard of progression or death (HR 1.37, 95% CI 1.12–1.69, *p* = 0.0028; C-index 0.672) (Table 4, Figure 2).

### 3.4. Prespecified Secondary Analysis: Median-Split Kaplan–Meier Analysis

**Figure 3:**
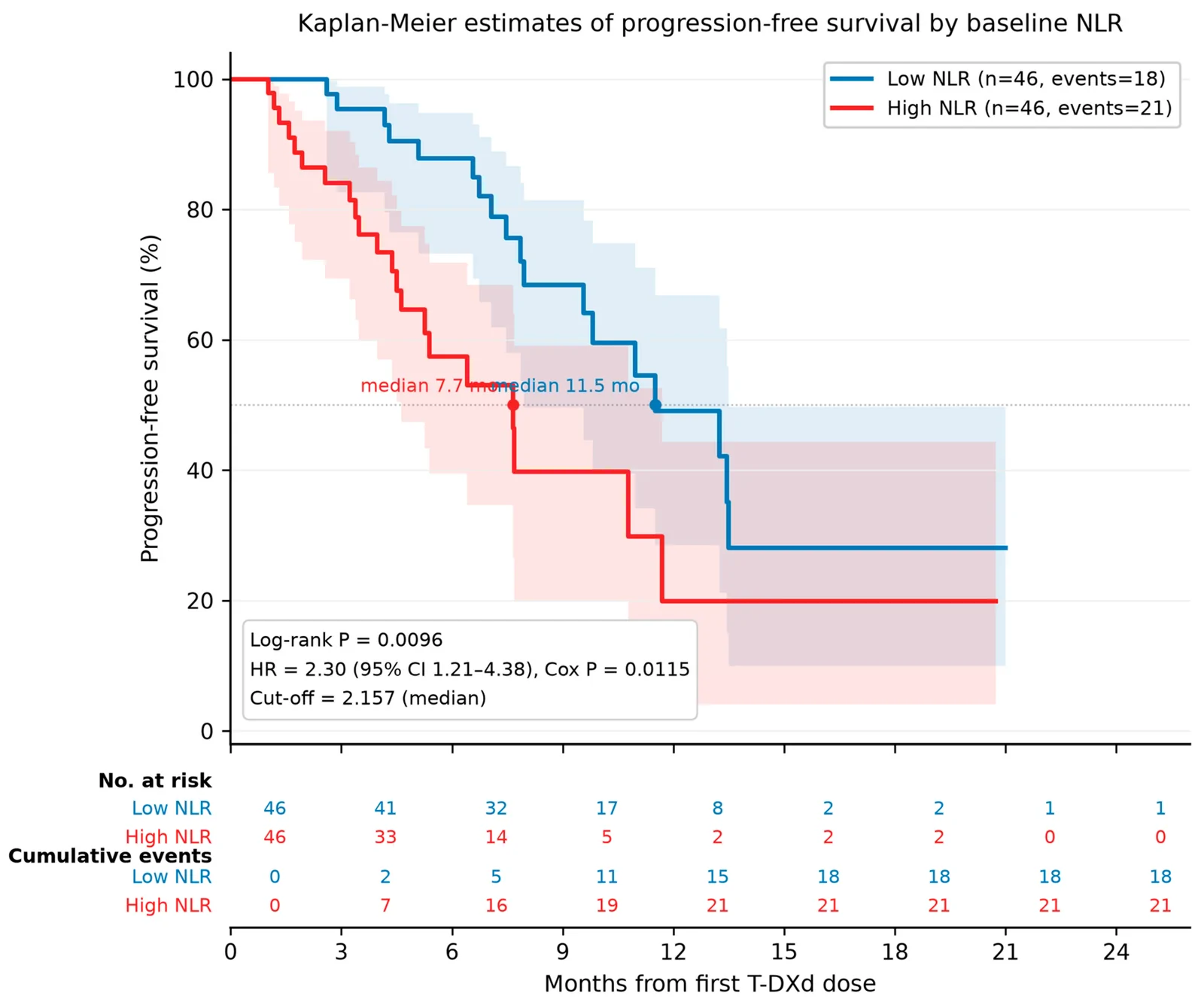
Kaplan–Meier curves for progression-free survival stratified by baseline NLR at the cohort median (2.157). Shaded bands denote 95% confidence intervals; the number at risk and cumulative events are shown below the axis. HR 2.30 (95% CI 1.21–4.38); log-rank *p* = 0.0096.

Using the cohort median (2.157) as the cut-off, median PFS was 7.66 months in the high-NLR group (n = 46, 21 events) and 11.50 months in the low-NLR group (n = 46, 18 events) (HR 2.30, 95% CI 1.21–4.38; log-rank *p* = 0.0096) (Figure 3). Six-month PFS rates were 57.5% (high) and 87.9% (low); 12-month rates were 19.9% and 49.1%, resting on 2 and 8 patients at risk, respectively.

### 3.5. Quartile Analysis

**Figure 4:**
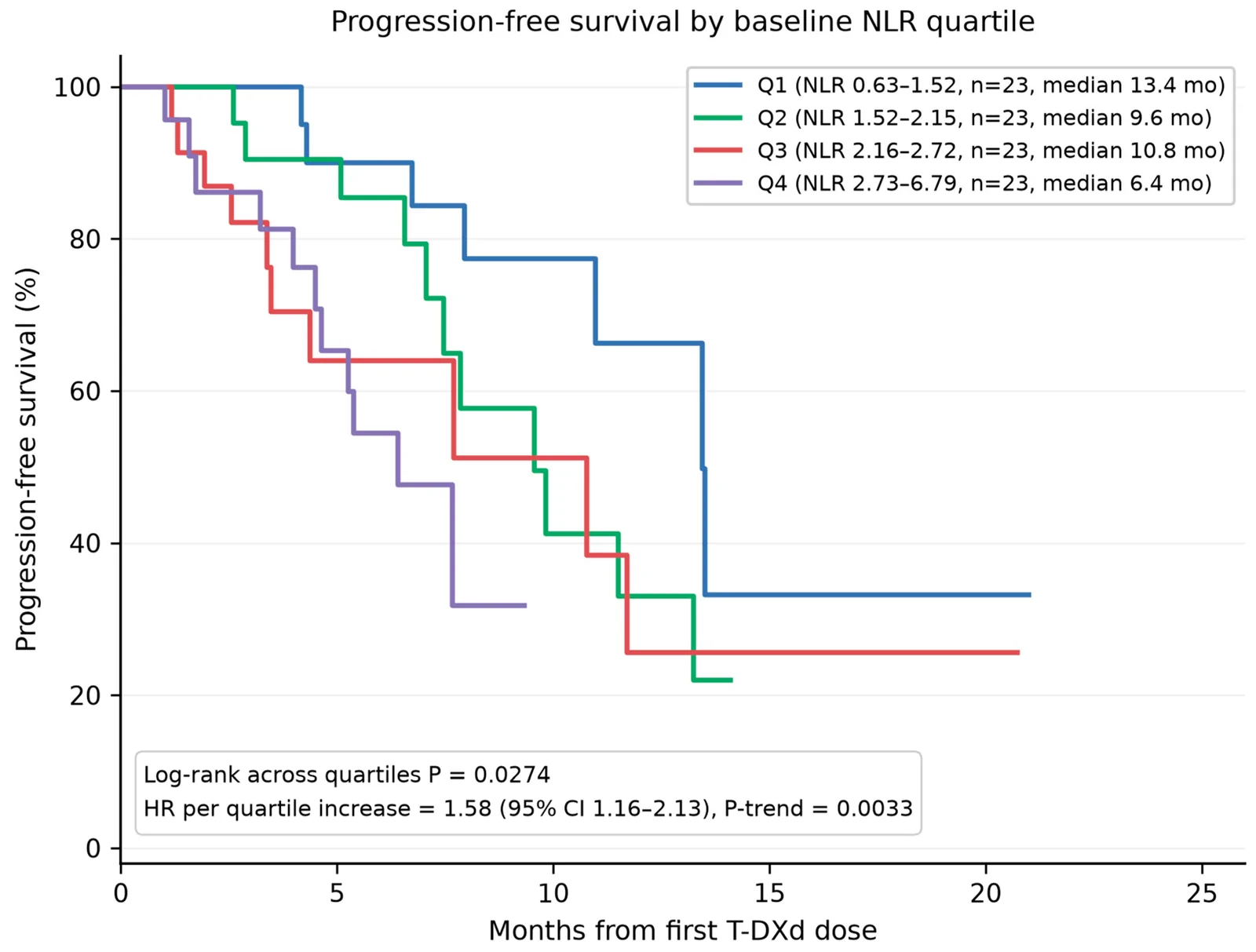
Kaplan–Meier curves for progression-free survival stratified by quartiles of baseline NLR. *p* for trend = 0.0033.

Quartile cut-offs were 1.52, 2.16 and 2.72. Median PFS was 13.44 months in Q1 (NLR 0.63–1.52), 9.56 in Q2 (1.52–2.15), 10.78 in Q3 (2.16–2.72) and 6.41 in Q4 (2.73–6.79). Relative to Q1, hazard ratios were 1.89 (95% CI 0.73–4.89) for Q2, 2.68 (1.02–7.09) for Q3 and 4.14 (1.52–11.26) for Q4; the trend test was significant (HR per quartile 1.58, 95% CI 1.16–2.13, *p* for trend = 0.0033; log-rank across quartiles *p* = 0.027) (Figure 4). Median PFS did not decrease monotonically across the middle quartiles (Q3 exceeded Q2), and we therefore report the trend as statistically significant without characterising it as monotonic.

### 3.6. Individual Blood Components

No individual component was significant as a continuous variable: neutrophil count HR 1.22 per 10^9^/L (95% CI 0.95–1.56, *p* = 0.12); lymphocyte count HR 0.60 per 10^9^/L (0.33–1.08, *p* = 0.091); platelet count HR 1.000 per 10^9^/L (0.996–1.003, *p* = 0.79); PLR HR 1.000 per unit (0.998–1.003, *p* = 0.73). At their medians, however, the lymphocyte count showed a significant association with PFS (log-rank *p* = 0.011), whereas neutrophils (*p* = 0.16), platelets (*p* = 0.61) and PLR (*p* = 0.26) did not (Table 5, Supplementary Figure S1).

### 3.7. Incremental Value of NLR over the Absolute Lymphocyte Count and over the Clinical Model

Because the lymphocyte count is a mathematical constituent of NLR, incremental value was quantified by nested-model comparison. A model containing the lymphocyte count alone had AIC 286.09 and C-index 0.638; a model containing NLR alone had AIC 282.04 and C-index 0.672; a model containing both had AIC 283.83 and C-index 0.674. Adding NLR to the lymphocyte count improved fit (likelihood-ratio χ^2^ = 4.25, df = 1, *p* = 0.039; ΔAIC −2.25; ΔC-index +0.036), whereas adding the lymphocyte count to NLR did not (χ^2^ = 0.21, df = 1, *p* = 0.65). For comparison, adding NLR to the neutrophil count likewise improved fit (AIC 286.89 to 283.77; C-index 0.563 to 0.661). Adding NLR to the clinically driven model (performance status, visceral metastasis, hormone receptor status and age; *n* = 92, 39 events) improved discrimination from C-index 0.604 to 0.682 and fit from AIC 290.08 to 286.01 (likelihood-ratio χ^2^ = 6.07, df = 1, *p* = 0.014). NLR therefore carries prognostic information beyond the lymphocyte count and beyond standard clinical variables, while the lymphocyte count adds none beyond NLR.

### 3.8. Univariable and Multivariable Analyses

In univariable analysis, continuous NLR (*p* = 0.0028) and dichotomised NLR (*p* = 0.011) reached *p* < 0.05, as did high Ki-67 (HR 0.32, 95% CI 0.11–0.92, *p* = 0.034)—although that estimate rests on only 7 patients in the Ki-67 < 20% reference category and is correspondingly imprecise. ECOG performance status ≥ 1 showed a non-significant adverse trend (HR 1.69, 95% CI 0.89–3.23, *p* = 0.11); no other covariate was significant (Table 4).

In the clinically driven multivariable model (NLR, ECOG performance status, visceral metastasis as recorded in the source dataset, hormone receptor status, age > 60; *n* = 92, 39 events, 7.8 events per variable), NLR remained an independent prognostic factor (HR 1.36 per unit, 95% CI 1.09–1.70, *p* = 0.0062); ECOG performance status ≥ 1 showed a non-significant adverse trend (HR 1.73, 0.90–3.32, *p* = 0.099), and the remaining covariates were not significant (Table 6). The minimal model (13.0 events per variable) gave a consistent estimate (HR 1.33, 1.08–1.65, *p* = 0.0086), as did the two extended models adding liver and brain involvement together with either prior anti-HER2 lines (*n* = 73, 35 events, 4.4 events per variable; HR 1.66, 1.16–2.37, *p* = 0.0051) or total treatment lines (*n* = 84, 36 events, 4.5 events per variable; HR 1.48, 1.14–1.93, *p* = 0.0033), and the ridge-penalised model (HR 1.31, 1.06–1.62, *p* = 0.0120); C-indices were 0.673, 0.726, 0.666 and 0.679 respectively (Table 6, Supplementary Figure S2).

### 3.9. Model Diagnostics and Sensitivity Analyses

**Figure 5:**
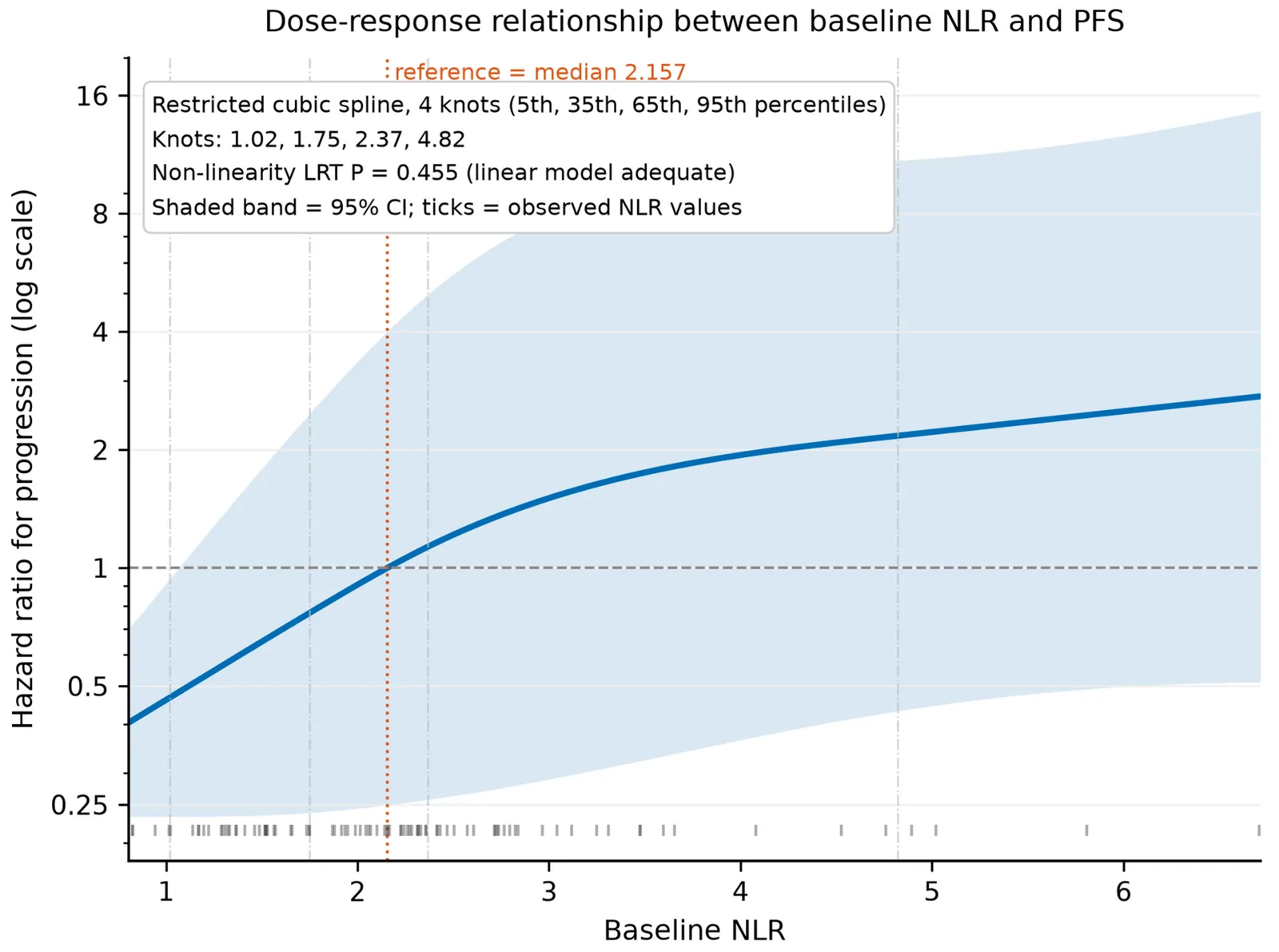
Restricted cubic spline estimate of the hazard ratio for progression-free survival across the observed range of baseline NLR, with pointwise 95% confidence bands. Knots at the 5th, 35th, 65th and 95th percentiles; reference value is the median NLR (2.157). Tick marks along the base show the distribution of observed values. Non-linearity test *p* = 0.46.

The proportional-hazards assumption was not violated for NLR (Schoenfeld residual test: rank transform *p* = 0.75, Kaplan–Meier transform *p* = 0.71, identity transform *p* = 0.67; median-split NLR *p* = 0.064) nor for any covariate of the clinically driven multivariable model (NLR *p* = 0.93, ECOG *p* = 0.43, visceral metastasis *p* = 0.98, hormone receptor status *p* = 0.72, age *p* = 0.92). Restricted cubic spline analysis with three, four and five knots showed no evidence of non-linearity in the association between NLR and the log hazard (four knots: likelihood-ratio χ^2^ = 1.57, df = 2, *p* = 0.46; three knots *p* = 0.33; five knots *p* = 0.63; quadratic term *p* = 0.071), and the linear specification was preferred on AIC in every comparison (282.17 versus 283.22, 284.59 and 286.43) (Figure 5).

The primary result was robust across prespecified sensitivity analyses: using the composite endpoint of progression or death (40 events), HR 1.40 (95% CI 1.14–1.71, *p* = 0.0011); using the alternative progression-status field recorded in the source dataset (*n* = 85, 33 events), HR 1.89 (1.46–2.44, *p* < 0.0001); modelling log(NLR), HR 2.93 per unit (1.49–5.74, *p* = 0.0018); restricting to NLR ≤ 4 (*n* = 84), HR 1.86 (1.07–3.24, *p* = 0.029); and relaxing the timing rule to exclude only candidates whose baseline count was drawn after the first dose (*n* = 103), HR 1.35 (1.13–1.61, *p* = 0.0010) (Supplementary Figure S3).

One prespecified sensitivity analysis warrants explicit discussion. Reinstating the single candidate excluded for an unreliable baseline value (lymphocyte count 0.04 ×10^9^/L, derived NLR 113.75) abolished the association on the linear scale (*n* = 93; HR 1.00 per unit, 95% CI 0.96–1.04, *p* = 0.83) but not under log(NLR) parametrisation (HR 1.48 per unit, 1.02–2.16, *p* = 0.041). This value acts as a leverage point on the linear scale: it is an extreme ratio derived from a lymphocyte count at the assay lower limit, its progression status is censored, and it is more than an order of magnitude above every other value in the cohort. We report this behaviour transparently rather than silently: it is the expected consequence of a single extreme observation in a linear specification, the log-scale result confirms that the underlying association is not an artefact of the exclusion, and the primary specification excludes the value on the prespecified plausibility rule of Section 2.3.

### 3.10. Exploratory Subgroup Analyses and Centre Analysis

The NLR–PFS association was directionally consistent across all reportable exploratory strata, reaching significance in most, including HR-positive patients (HR 1.34, 95% CI 1.05–1.73, *p* = 0.021), patients with visceral metastasis by the source-record field (1.29, 1.02–1.64, *p* = 0.036) and by the reconstructed liver/lung/brain definition (1.35, 1.09–1.67, *p* = 0.0068), patients without liver metastasis (1.52, 1.14–2.03, *p* = 0.0047), patients without brain metastasis (1.86, 1.20–2.88, *p* = 0.0054), patients with lung metastasis (1.65, 1.20–2.27, *p* = 0.0021), patients aged 60 years or younger (1.79, 1.25–2.56, *p* = 0.0014), patients with more than two prior anti-HER2 lines (2.13, 1.46–3.09, *p* < 0.0001), patients with more than two total treatment lines (1.39, 1.09–1.78, *p* = 0.0092), patients with fewer than three metastatic sites (1.59, 1.23–2.06, *p* = 0.0004), patients with BMI ≥ 25 (1.89, 1.34–2.68, *p* = 0.0003), patients with Ki-67 ≥ 20% (1.29, 1.02–1.64, *p* = 0.036) and patients with de novo stage IV disease (1.86, 1.18–2.93, *p* = 0.0071) (Table 2, Supplementary Figure S4). Each stratum is restricted to patients with the stratifying variable recorded, and its denominator is shown; one stratum was not reportable because it contained fewer than 15 patients (Ki-67 < 20%, *n* = 7, 4 events).

Two further specifications are reported because they bear directly on interpretation. First, a model retaining DFS category could be fitted only in the 57 patients with an evaluable disease-free interval (24 events, 4.0 events per variable); in that complete-case model, NLR was no longer significant (HR 1.24, 95% CI 0.95–1.62, *p* = 0.106) and DFS category itself was not prognostic (HR 1.03, 0.41–2.61, *p* = 0.95). We therefore report the model without DFS as the primary multivariable analysis and present the DFS-containing model as a sensitivity analysis, stating plainly that its loss of significance reflects the 38% reduction in sample size and the correspondingly low events-per-variable ratio rather than evidence against the association. Second, substituting the reconstructed visceral-metastasis definition (liver, lung or brain) for the source-record field left the NLR estimate essentially unchanged (HR 1.39, 1.13–1.73, *p* = 0.0023), confirming that the primary estimate is not sensitive to how visceral metastasis is coded.

No interaction reached significance, either before or after multiplicity correction. Across the seventeen exposure-by-subgroup interaction tests, none was nominally significant at *p* < 0.05 (smallest raw *p* = 0.052 for prior anti-HER2 treatment lines, with all Benjamini–Hochberg adjusted q values ≥ 0.372). The apparent differences between strata—most notably the stronger point estimate in patients with more than two prior anti-HER2 lines (HR 2.13) than in those with at most two (HR 1.20), and in patients with BMI ≥ 25 (HR 1.89) than in those with BMI < 25 (HR 1.14)—are therefore not evidence of effect heterogeneity and are reported as such. We note explicitly that an earlier draft of this manuscript had reported a nominally significant interaction for prior anti-HER2 lines; that finding was an artefact of the missing-data coding error described in Section 2.6, under which 19 patients with unrecorded treatment lines had been assigned to the ≤2 lines reference category. Once those patients are correctly excluded from the stratification, the interaction is no longer significant. Given the exploratory nature of these analyses, they are reported for completeness and hypothesis generation only, and no subgroup-restricted recommendation is made.

The centre-stratified analysis showed no material centre effect: the hazard ratio for PFS at Beijing Friendship Hospital relative to Peking University Cancer Hospital was 1.14 (95% CI 0.54–2.41, *p* = 0.73); baseline NLR distributions were similar between the two centres (Peking University Cancer Hospital median 2.19; Beijing Friendship Hospital median 2.09); the NLR–PFS association adjusted for centre was unchanged (HR 1.37, 95% CI 1.11–1.69, *p* = 0.0029); and the association was directionally consistent within each centre (Peking University Cancer Hospital: HR 1.38, 1.11–1.72, *p* = 0.0037; Beijing Friendship Hospital: HR 1.19, 0.62–2.29, *p* = 0.61), with a non-significant NLR-by-centre interaction (*p* = 0.73).

## 4. Discussion

In this two-centre real-world cohort of 92 patients with HER2-positive advanced breast cancer treated with T-DXd, elevated baseline NLR was an independent adverse prognostic marker for progression-free survival. The association was significant as a continuous variable (HR 1.37 per unit, *p* = 0.0028), at the cohort median cut-off (HR 2.30, log-rank *p* = 0.0096), across quartiles with a significant trend (*p* = 0.0033), in the clinically driven multivariable model and in a minimal model, two extended models and a ridge-penalised model, beyond the absolute lymphocyte count and beyond the clinical covariate set, in seven sensitivity analyses, and consistently across both contributing centres; model diagnostics confirmed that the proportional-hazards and linearity assumptions were satisfied. The finding is consistent with the prognostic role of baseline NLR reported in trastuzumab-treated HER2-positive disease, including exploratory analyses of the CLEOPATRA trial [20] and independent cohorts [21,22], and extends it to the ADC era.

Three aspects of the analysis deserve emphasis because they address methodological concerns raised in this literature. First, the incremental value of NLR was tested against its own mathematical constituent rather than asserted: adding NLR to the lymphocyte count significantly improved model fit (likelihood-ratio *p* = 0.039, ΔAIC −2.25, ΔC-index +0.036), whereas the reverse comparison did not (*p* = 0.65); adding NLR to the clinically driven covariate set improved discrimination from a C-index of 0.604 to 0.682 (likelihood-ratio *p* = 0.014). The prognostic signal is therefore carried by the composite ratio and not reducible to the lymphocyte count alone, although the median-split association of the lymphocyte count (log-rank *p* = 0.011) indicates that lymphopenia is itself a meaningful adverse signal—a pattern consistent with the biological interpretation that NLR reflects the balance between pro-tumour inflammation and impaired adaptive immune surveillance [7,8,23]. Second, the timing rule that defines a baseline blood count was applied uniformly to every screened candidate and every exclusion is reported with its count and failure mode, including the 16 enrolled-type candidates whose counts were drawn after the first dose and the 17 drawn more than 7 days before it; the median cut-off was fixed at the median of the analysis cohort and reported with its derivation. Third, the quartile analysis is reported with per-quartile hazard ratios relative to Q1 and an explicit trend test; because median PFS was not monotonic across the middle quartiles, no monotonic dose–response is claimed.

We also report, in the interest of full transparency, a data-handling error identified and corrected during this revision. An earlier draft had entered several binary covariates using pre-computed indicator fields from the source dataset in which patients with a missing or undefined source value had been recoded into one of the two categories rather than excluded—most consequentially, the 35 patients without an evaluable disease-free interval had been coded as “DFS ≥ 2 years” and the 19 patients with unrecorded prior anti-HER2 treatment lines as “≤2 lines”. Because these recodings placed patients with unknown values into a substantive category, they produced denominators inconsistent with the reported availability of each variable. All binary covariates have been re-derived from their source variables, patients with missing values are now excluded from the analyses containing the relevant covariate (complete-case, per-covariate), and every affected estimate has been recomputed. This correction did not alter the primary result—baseline NLR was complete in all 92 patients, and its hazard ratio is unchanged—but it did change two findings that we now report honestly rather than retain: the multivariable model was re-specified without DFS (which is evaluable in only 57 patients), and the interaction for prior anti-HER2 treatment lines, which had appeared significant in the earlier draft, is no longer significant once the 19 patients with unrecorded treatment lines are correctly excluded from that stratification.

No subgroup interaction was statistically significant, before or after Benjamini–Hochberg correction across the seventeen tests performed (smallest raw *p* = 0.052 for prior anti-HER2 treatment lines; all adjusted q ≥ 0.372). We therefore make no claim of effect heterogeneity and offer no subgroup-restricted interpretation. The point estimates nonetheless show a pattern worth naming as a hypothesis for future work rather than as a finding: the association appeared numerically stronger in patients with more than two prior anti-HER2 treatment lines (HR 2.13) than in those with at most two (HR 1.20), and in patients with BMI ≥ 25 (HR 1.89) than in those with BMI < 25 (HR 1.14). Both comparisons rest on small strata—17 and 31 patients respectively in the smaller arm—and neither survives multiplicity correction, so neither supports a claim that NLR behaves differently in these groups. It is worth recording that the anti-HER2-line comparison did appear significant in an earlier draft of this work (interaction *p* = 0.0033); as explained above, that result was an artefact of coding 19 patients with unrecorded treatment lines into the ≤2 lines reference category, and it dissipated once those patients were correctly excluded. We report this reversal explicitly because it illustrates how readily a spurious interaction can arise from missing-data recoding in a cohort of this size, and because an uncorrected claim of effect modification by treatment line would have been the most clinically consequential error in the manuscript.

Our findings should be interpreted against the mechanism of ADC action; unlike monoclonal antibodies, whose activity partly depends on Fc-mediated immune effector functions, T-DXd acts through target-mediated internalisation and intracellular payload release. That baseline systemic inflammation retains prognostic value under ADC therapy suggests that the inflammatory milieu reflects tumour biology and host reserve more than it reflects the immune effector mechanisms of any single drug class—a conclusion consistent with the prognostic role of NLR across diverse treatment settings [9,10,11,12,13]. Conversely, the absence of prognostic utility reported for NLR in early breast cancer [26] underscores that its value is stage- and context-dependent, and our cohort—heavily pretreated advanced disease, median three prior anti-HER2 lines—represents a context in which host factors plausibly dominate.

The study has limitations, stated plainly. First, the design is retrospective and the cohort modest: 92 patients and 39 events give limited precision for subgroup and landmark estimates, and the 12- and 18-month rates rest on 10 and 4 patients at risk respectively, with correspondingly wide confidence intervals. Second, death ascertainment was incomplete (two deaths recorded, one of which coincided with progression), so PFS was in practice progression-driven, overall survival could not be analysed, and the composite endpoint—although giving a consistent result—cannot fully substitute for complete vital-status ascertainment. Third, corticosteroid and G-CSF exposure, active infection and the interval since the last prior systemic therapy were not captured; each can alter neutrophil and lymphocyte counts within days, and residual confounding of the exposure itself cannot be excluded. Fourth, the centre was identified only by the format of the anonymised identifier rather than by an explicit centre field; the centre-stratified analysis showed no material centre effect and a non-significant NLR-by-centre interaction, but Beijing Friendship Hospital contributed only 18 patients, and the analysis is underpowered to exclude modest centre-level differences in patient selection, dosing practice or imaging schedule. Fifth, one screened candidate was excluded under the prespecified plausibility rule for a baseline lymphocyte count at the assay lower limit; the sensitivity analysis reinstating this value shows that it acts as a leverage point on the linear scale, and although the log-scale result confirms that the association is not an artefact of the exclusion, the behaviour of a single extreme observation in a 92-patient cohort is itself a limitation of precision. Sixth, NLR was measured once at baseline; longitudinal kinetics, which may carry additional information, were not analysed. Seventh, the DFS variable was evaluable in only 57 patients because de novo metastatic presentation (*n* = 28) leaves the disease-free interval undefined, and the DFS coefficient in the multivariable model should be read accordingly. Eighth, the cohort is drawn from two Chinese centres and generalisability to other populations is unestablished. Ninth, because every patient received T-DXd and no comparator arm exists, NLR is a prognostic marker of outcome among treated patients and not a predictive biomarker of differential T-DXd benefit; no statement regarding treatment selection is made. Tenth, the composite visceral-metastasis variable as recorded in the source dataset disagrees with the union of the individually verified site-specific variables in 18 of 92 patients (see the footnote to Table 1); we retained the source variable for consistency with the dataset as supplied and report the discrepancy rather than silently re-deriving it, and note that visceral metastasis was non-significant in every multivariable specification, so the primary NLR estimate is not sensitive to how this covariate is coded.

Within these limits, the findings carry two implications. For research, they establish baseline NLR as a candidate prognostic biomarker in T-DXd-treated HER2-positive advanced breast cancer and identify testable hypotheses—notably whether the association differs by the intensity of prior anti-HER2 treatment or by body composition, neither of which this cohort had power to resolve, and the comparison of inflammatory prognostication across antibody and ADC therapy—that require prospective cohorts or randomised-trial cohorts with stored baseline counts. For practice, baseline NLR is simple, inexpensive and universally available, and may support risk stratification and monitoring intensity in this population; we emphasise that prospective validation with standardised sampling, complete vital-status ascertainment and, ideally, external replication is required before any threshold-based clinical application.

## 5. Conclusions

In this two-centre real-world cohort of patients with HER2-positive advanced breast cancer treated with trastuzumab deruxtecan, elevated baseline neutrophil-to-lymphocyte ratio was an independent adverse prognostic marker for progression-free survival, analysed robustly as a continuous variable, with a significant quartile trend and a concordant median-split separation, carrying prognostic information beyond the absolute lymphocyte count and beyond standard clinical variables, and consistent across both contributing centres. Baseline NLR is simple, inexpensive and routinely available and may support clinical risk stratification and monitoring intensity in this setting. Prospective, multicentre validation with standardised sampling and complete death ascertainment is required before any actionable threshold can be defined.

## Figures and Tables

**Table 1:** Baseline characteristics of the analysis cohort (*n* = 92).

Characteristic	Value
Age, years—median (IQR), range	60.5 (53–66), 34–78
Age > 60 years, *n* (%)	46 (50.0)
ECOG performance status, n—0/1/2/3	53/34/4/1
ECOG PS ≥ 1, *n* (%)	39 (42.4)
BMI, kg/m^2^—median (IQR), range	23.69 (20.41–26.13), 16.23–37.18 (*n* = 84)
BMI ≥ 25 kg/m^2^, *n* (%)	31 (36.9) of 84 with data
Hormone receptor-positive, *n* (%)	58 (63.0)
Visceral metastasis (source-record field), *n* (%)	81 (88.0)
Visceral metastasis (liver, lung or brain), *n* (%)	77 (83.7)
Liver metastasis, *n* (%)	43 (46.7)
Lung metastasis, *n* (%)	39 (42.4)
Brain metastasis, *n* (%)	39 (42.4)
Metastatic sites ≥ 2, *n* (%)	50 (54.3)
Metastatic sites ≥ 3, *n* (%)	25 (27.2)
De novo stage IV, *n* (%)	28 (30.4)
Ki-67 ≥ 20%, *n* (%)	76 (91.6) of 83 with data
DFS, months—median (IQR), range	24 (14–43), 5–84 (*n* = 57)
DFS ≥ 2 years, *n* (%)	29 (50.9) of 57 with data
Treatment line at which T-DXd was given—median (IQR), range	4 (3–5), 2–14 (*n* = 84)
—2nd/3rd/≥4th line, *n* (%)	20 (23.8)/21 (25.0)/43 (51.2)
Total treatment lines > 2, *n* (%)	64 (76.2) of 84 with data
Prior anti-HER2 treatment lines—median (IQR), range	3 (3–4), 2–14 (*n* = 73)
Prior anti-HER2 lines > 2, *n* (%)	56 (76.7) of 73 with data
Prior salvage chemotherapy lines—median (IQR), range	2 (1–3), 0–8 (*n* = 91)
Prior endocrine lines for metastatic disease—median (IQR), range	0 (0–1), 0–5 (*n* = 88)
Prior trastuzumab, *n* (%)	75 (94.9) of 79 with data
Prior pertuzumab, *n* (%)	65 (83.3) of 78 with data
Prior tyrosine kinase inhibitor, *n* (%)	65 (82.3) of 79 with data
Prior T-DM1, *n* (%)	26 (35.6) of 73 with data
Neutrophils, ×10^9^/L—median (IQR), range	2.88 (2.34–3.99), 0.55–7.70
Lymphocytes, ×10^9^/L—median (IQR), range	1.43 (1.03–1.84), 0.42–3.77
Platelets, ×10^9^/L—median (IQR), range	218 (166–273), 72–535
NLR—median (IQR), range	2.16 (1.52–2.72), 0.63–6.79
PLR—median (IQR), range	144.3 (108.0–209.1), 34.4–851.3
PLR > 144, *n* (%)	48 (52.2)
Study centre—Peking University Cancer Hospital/Beijing Friendship Hospital, *n* (%)	74 (80.4)/18 (19.6)
T-DXd initiation—first/last date	14 January 2023/25 June 2025
Data cutoff	16 Oct 2025
Deaths recorded during follow-up, n	2

Note 1 (visceral metastasis): the source-record visceral-metastasis count (81, 88.0%) is the categorical variable as recorded in the source dataset and is not the arithmetic union of the site-specific variables; the union of liver, lung and brain involvement comprises 77 patients (83.7%). The two differ in 18 patients (11 recorded as visceral-positive with only nodal, chest-wall or cutaneous involvement, and 7 recorded as visceral-negative despite liver, lung or brain involvement). The site-specific variables were each verified against the source metastatic-site text field and were concordant in 92/92 patients. Both definitions are carried through the analyses, and Section 3.8 reports that the multivariable NLR estimate is unchanged when the reconstructed definition is substituted. Note 2 (denominators): every binary covariate was re-derived from its source variable at the time of analysis, and patients with a missing or undefined source value are excluded from the analyses containing that covariate rather than being assigned to either category. DFS is undefined for the 28 patients with de novo metastatic presentation and unrecorded for 7 others (evaluable *n* = 57); prior anti-HER2 treatment lines were unrecorded for 19 patients (*n* = 73); Ki-67 for 9 (*n* = 83); height and weight, from which BMI was computed, for 8 (*n* = 84); and total treatment lines for 8 (*n* = 84). Age, ECOG performance status, hormone receptor status, metastatic-site variables, all blood indices and NLR were complete in all 92 patients.Abbreviations: BMI, body mass index; DFS, disease-free survival; ECOG PS, Eastern Cooperative Oncology Group performance status; HER2, human epidermal growth factor receptor 2; IQR, interquartile range; Ki-67, proliferation marker Ki-67; NLR, neutrophil-to-lymphocyte ratio; PLR, platelet-to-lymphocyte ratio; T-DM1, trastuzumab emtansine; T-DXd, trastuzumab deruxtecan. ‘De novo stage IV’ denotes American Joint Committee on Cancer stage IV disease present at initial diagnosis. In the treatment-line rows, an em dash (—) marks an indented subcategory of the row immediately above (for example, ‘—2nd/3rd/≥4th line’ enumerates the line at which T-DXd was given).

**Table 2:** Exploratory subgroup analyses of the NLR–PFS association, with interaction testing (raw and FDR-adjusted).

Subgroup	n/Evaluable	Events	HR per Unit NLR	95% CI	*p*	*p* Interaction	FDR q
Hormone receptor status						0.837	0.923
HR-positive	58/92	26	1.34	1.05–1.73	0.021		
HR-negative	34/92	13	1.42	0.99–2.03	0.057		
Visceral metastasis (source-record field)						0.133	0.372
Visceral metastasis	81/92	35	1.29	1.02–1.64	0.036		
Visceral metastasis (liver, lung or brain)						0.186	0.372
Visceral metastasis	77/92	34	1.35	1.09–1.67	0.0068		
No visceral metastasis	15/92	5	2.34	0.88–6.20	0.088		
Liver metastasis						0.202	0.372
Liver metastasis	43/92	19	1.24	0.91–1.69	0.18		
No liver metastasis	49/92	20	1.52	1.14–2.03	0.0047		
Lung metastasis						0.330	0.431
Lung metastasis	39/92	18	1.65	1.20–2.27	0.0021		
No lung metastasis	53/92	21	1.28	0.94–1.75	0.12		
Brain metastasis						0.219	0.372
Brain metastasis	39/92	18	1.29	0.99–1.70	0.062		
No brain metastasis	53/92	21	1.86	1.20–2.88	0.0054		
Body-mass index						0.101	0.372
BMI < 25	53/84	20	1.14	0.83–1.57	0.41		
BMI ≥ 25	31/84	16	1.89	1.33–2.68	0.0003		
Age						0.094	0.372
Age ≤ 60	46/92	20	1.79	1.25–2.56	0.0014		
Age > 60	46/92	19	1.21	0.90–1.63	0.21		
ECOG performance status						0.310	0.431
ECOG 0	53/92	16	1.54	1.11–2.15	0.010		
ECOG ≥ 1	39/92	23	1.28	0.97–1.68	0.085		
Disease-free survival category						0.905	0.923
DFS < 2 years	28/57	12	1.45	0.93–2.25	0.10		
DFS ≥ 2 years	29/57	12	1.21	0.86–1.68	0.27		
Prior anti-HER2 treatment lines						0.052	0.372
Prior anti-HER2 lines ≤ 2	17/73	10	1.20	0.79–1.82	0.40		
Prior anti-HER2 lines > 2	56/73	25	2.13	1.46–3.09	< 0.0001		
Total treatment lines						0.923	0.923
Total treatment lines ≤ 2	20/84	9	1.28	0.86–1.91	0.22		
Total treatment lines > 2	64/84	27	1.39	1.08–1.78	0.0092		
Number of metastatic sites (≥3)						0.111	0.372
Metastatic sites < 3	67/92	28	1.59	1.23–2.06	0.0004		
Metastatic sites ≥ 3	25/92	11	1.07	0.68–1.70	0.77		
Number of metastatic sites (≥2)						0.217	0.372
Metastatic sites < 2	42/92	16	1.89	1.03–3.45	0.038		
Metastatic sites ≥ 2	50/92	23	1.36	1.06–1.73	0.014		
Ki-67						0.092	0.372
Ki-67 ≥ 20%	76/83	30	1.29	1.02–1.64	0.036		
De novo stage IV						0.302	0.431
De novo stage IV	28/92	14	1.86	1.18–2.93	0.0071		
Not de novo stage IV	64/92	25	1.25	0.97–1.61	0.088		
Study centre						0.727	0.882
Peking University Cancer Hospital	74/92	30	1.38	1.11–1.72	0.0037		
Beijing Friendship Hospital	18/92	9	1.19	0.61–2.29	0.61		

Exploratory; reported for completeness and hypothesis generation only. Subgroup-specific *p*-values are not interpreted as evidence of effect heterogeneity. Every binary stratifying variable was re-derived from its source variable, and the “n/evaluable” column shows the number of patients in the stratum over the number with that variable recorded; patients with a missing or undefined value are excluded from the stratum rather than assigned to a category. Strata with fewer than 15 patients or fewer than 5 events are not reported: Ki-67 < 20% (*n* = 7, 4 events). “*p* interaction” is the exposure-by-subgroup product-term *p*-value from a Cox model on all patients evaluable for that variable; “FDR q” is the Benjamini–Hochberg adjusted value across the seventeen interaction tests (Bonferroni-corrected threshold 0.0029). No interaction was significant before or after correction (smallest raw *p* = 0.052; all q ≥ 0.372).

**Table 3:** Landmark progression-free survival rates with confidence intervals, patients at risk and cumulative events.

Landmark	PFS Rate, %	95% CI, %	Patients at Risk	Cumulative Events
3 months	89.8	81.3–94.6	74	9
6 months	73.7	62.3–82.1	46	21
9 months	55.7	42.1–67.3	22	30
12 months	37.1	22.6–51.6	10	36
18 months	24.7	11.5–40.6	4	39
24 months	24.7	11.5–40.6	1	39

Median follow-up by reverse Kaplan–Meier: 7.6 months (IQR 4.3–8.5 among censored patients). The 12-, 18- and 24-month estimates rest on small risk sets and should be interpreted with caution.

**Table 4:** Univariable Cox proportional-hazards analyses of progression-free survival (*n* = 92 unless stated).

Variable	n	HR	95% CI	*p*
NLR (per unit increase)	92	1.37	1.12–1.69	0.0028
High NLR (>2.157) vs. low	92	2.30	1.21–4.38	0.011
Lymphocyte count (per 10^9^/L)	92	0.60	0.33–1.08	0.091
Neutrophil count (per 10^9^/L)	92	1.22	0.95–1.56	0.12
Platelet count (per 10^9^/L)	92	1.000	0.996–1.003	0.79
PLR (per unit increase)	92	1.000	0.998–1.003	0.73
High PLR (>144) vs. low	92	1.45	0.77–2.73	0.25
Age (per year)	92	0.99	0.96–1.01	0.33
Age > 60 vs. ≤60	92	0.84	0.45–1.58	0.59
ECOG PS ≥ 1 vs. 0	92	1.69	0.89–3.23	0.11
BMI ≥ 25 vs. <25	84	1.01	0.52–1.96	0.98
HR-positive vs. negative	92	0.90	0.46–1.77	0.77
Visceral metastasis (source-record field) vs. none	92	0.49	0.17–1.43	0.19
Visceral metastasis (liver, lung or brain) vs. none	92	1.21	0.47–3.09	0.70
Liver metastasis vs. none	92	0.94	0.50–1.77	0.85
Lung metastasis vs. none	92	1.36	0.72–2.56	0.35
Brain metastasis vs. none	92	1.10	0.59–2.07	0.77
Metastatic sites ≥ 2 vs. <2	92	0.98	0.52–1.86	0.95
Metastatic sites ≥ 3 vs. <3	92	1.02	0.51–2.06	0.95
DFS (per month)	57	0.99	0.97–1.01	0.38
DFS ≥ 2 years vs. <2	57	1.04	0.47–2.34	0.92
Prior anti-HER2 lines > 2 vs. ≤2	73	0.60	0.29–1.26	0.18
Total treatment lines > 2 vs. ≤2	84	0.86	0.41–1.84	0.71
Ki-67 ≥ 20% vs. <20%	83	0.32	0.11–0.91	0.034
De novo stage IV vs. no	92	1.14	0.59–2.21	0.69

HR, hazard ratio; CI, confidence interval; NLR, neutrophil-to-lymphocyte ratio; PLR, platelet-to-lymphocyte ratio; DFS, disease-free survival; ECOG PS, Eastern Cooperative Oncology Group performance status. The n column is the number of patients evaluable for that covariate: patients with a missing or undefined source value are excluded from the corresponding analysis rather than being assigned to a category (DFS *n* = 57; prior anti-HER2 lines *n* = 73; total treatment lines and BMI *n* = 84; Ki-67 *n* = 83; all other covariates and the primary exposure complete in all 92).

**Table 5:** Individual blood components and progression-free survival.

Component	n	HR (Continuous)	95% CI	*p*	Median Cut-Off	Median-Split Log-Rank *p*
Neutrophil count (per 10^9^/L)	92	1.216	0.948–1.560	0.12	2.89	0.16
Lymphocyte count (per 10^9^/L)	92	0.601	0.333–1.084	0.091	1.43	0.011
Platelet count (per 10^9^/L)	92	1.000	0.996–1.003	0.79	218.0	0.61
PLR (per unit)	92	1.000	0.998–1.003	0.73	144.3	0.26
NLR (per unit)	92	1.373	1.115–1.691	0.0028	2.16	0.0096

**Table 6:** Multivariable Cox proportional-hazards models of progression-free survival (*n* = 92, 39 events).

Model and Term	EPV	HR	95% CI	*p*
Clinically driven model (primary multivariable analysis); *n* = 92, 39 events; C-index 0.682, AIC 286.0	7.8			
—NLR (per unit)		1.36	1.09–1.70	0.0062
—ECOG PS ≥ 1 vs. 0		1.73	0.90–3.32	0.099
—Visceral metastasis (source field) vs. none		0.63	0.20–1.99	0.44
—HR-positive vs. negative		0.81	0.40–1.64	0.56
—Age > 60 vs. ≤60		0.81	0.41–1.57	0.53
Minimal model; C-index 0.673, AIC 283.0	13.0			
—NLR (per unit)		1.33	1.08–1.65	0.0086
—ECOG PS ≥ 1 vs. 0		1.71	0.89–3.28	0.11
—Visceral metastasis (source field) vs. none		0.62	0.20–1.88	0.40
Extended model (adds liver, brain, prior anti-HER2 lines); *n* = 73, 35 events; C-index 0.726, AIC 242.0	4.4			
—NLR (per unit)		1.66	1.16–2.37	0.0051
—ECOG PS ≥ 1 vs. 0		2.32	1.07–5.01	0.032
—Visceral metastasis (source field) vs. none		0.85	0.14–5.26	0.87
—HR-positive vs. negative		0.84	0.36–1.97	0.69
—Age > 60 vs. ≤60		0.80	0.36–1.79	0.59
—Liver metastasis vs. none		0.95	0.47–1.94	0.89
—Brain metastasis vs. none		0.87	0.33–2.27	0.77
—Prior anti-HER2 lines > 2 vs. ≤2		0.60	0.23–1.58	0.30
Extended model (adds liver, brain, total treatment lines); *n* = 84, 36 events; C-index 0.666, AIC 260.2	4.5			
—NLR (per unit)		1.48	1.14–1.93	0.0033
—ECOG PS ≥ 1 vs. 0		2.20	1.02–4.74	0.045
—Visceral metastasis (source field) vs. none		0.98	0.25–3.89	0.97
—HR-positive vs. negative		0.74	0.34–1.64	0.46
—Age > 60 vs. ≤60		0.79	0.37–1.67	0.53
—Liver metastasis vs. none		0.71	0.35–1.44	0.34
—Brain metastasis vs. none		0.58	0.24–1.43	0.24
—Total treatment lines > 2 vs. ≤2		0.85	0.36–1.99	0.71
Sensitivity: model retaining DFS category (complete cases); *n* = 57, 24 events; C-index 0.686, AIC 166.3	4.0			
—NLR (per unit)		1.24	0.95–1.62	0.106
—ECOG PS ≥ 1 vs. 0		2.30	0.96–5.51	0.061
—Visceral metastasis (source field) vs. none		0.73	0.15–3.50	0.69
—HR-positive vs. negative		0.95	0.35–2.61	0.93
—Age > 60 vs. ≤60		0.68	0.27–1.74	0.42
—DFS ≥ 2 years vs. <2		1.03	0.41–2.61	0.95
Sensitivity: primary model with reconstructed visceral definition (liver, lung or brain); *n* = 92, 39 events; C-index 0.682, AIC 286.5	7.8			
—NLR (per unit)		1.39	1.13–1.73	0.0023
—ECOG PS ≥ 1 vs. 0		1.74	0.89–3.39	0.11
—Visceral metastasis (liver/lung/brain) vs. none		0.87	0.32–2.35	0.78
—HR-positive vs. negative		0.84	0.42–1.68	0.62
—Age > 60 vs. ≤60		0.76	0.39–1.47	0.41
Sensitivity: ridge-penalised clinically driven model (L2, penaliser 0.1); *n* = 92, 39 events; C-index 0.679, AIC 287.9	7.8			
—NLR (per unit)		1.31	1.06–1.62	0.012
—ECOG PS ≥ 1 vs. 0		1.55	0.87–2.77	0.14
—Visceral metastasis (source field) vs. none		0.69	0.25–1.89	0.47
—HR-positive vs. negative		0.86	0.46–1.60	0.63
—Age > 60 vs. ≤60		0.84	0.47–1.51	0.57

EPV, events per variable; AIC, Akaike information criterion; n and events are those evaluable for the covariates in each model. The clinically driven model was selected on clinical grounds rather than on univariate significance; it is described as clinically driven rather than prespecified because its covariate set was established during revision in response to peer review. DFS category was deliberately omitted from the primary model because it is evaluable in only 57 of 92 patients; the model retaining it is reported as a sensitivity analysis. Ridge penalisation 0.1 (L2). A dash (—) indicates that the covariate was not included in the corresponding model.

## Data Availability

The de-identified dataset supporting the conclusions of this article, together with the analysis code, is not publicly deposited. They are available from the corresponding author on reasonable request, subject to approval by the Institutional Review Boards of Peking University Cancer Hospital and Beijing Friendship Hospital. Public deposition is precluded because the data derive from patient medical records and their release is restricted by institutional data-governance policy and by patient-privacy requirements, notwithstanding de-identification.

## Supplementary Materials

The following supporting information can be downloaded at: https://www.mdpi.com/article/10.3390/jcm15218437/s1, Table S1: Variable definitions with coding, reference categories and available sample sizes; Table S2: Proportional-hazards test results for all model terms; Table S3: Restricted cubic spline coefficients and knot placements; Figure S1: Kaplan–Meier curves for progression-free survival stratified at the median of each individual blood component: neutrophil count, lymphocyte count, platelet count, platelet-to-lymphocyte ratio and NLR. Only the lymphocyte count and NLR showed a significant median-split association (log-rank *p* = 0.011 and 0.0096, respectively); Figure S2: Model fit (AIC) and discrimination (Harrell’s C-index) across modelling strategies. NLR alone and NLR added to the clinical model outperform every single blood component and the clinical model without NLR. Figure S3: (A) Distribution of baseline NLR with the median cut-off and interquartile and decile boundaries. (B) Sensitivity analyses of the primary endpoint: composite endpoint, alternative event definition, log(NLR), restriction to NLR ≤ 4, the relaxed timing window, and reinstatement of the single candidate with an unreliable baseline value on both the linear and log scales. The linear-scale estimate is sensitive to that single leverage point; the log-scale estimate is not; Figure S4: Forest plot of exploratory subgroup analyses of the NLR–PFS association, with hazard ratios, 95% confidence intervals, subgroup *p*-values, interaction *p*-values and the number of patients and events per stratum. No interaction was significant before or after Benjamini–Hochberg correction across the seventeen tests (smallest raw *p* = 0.052; all q ≥ 0.372).
