# Supplementary material for: Prognostic Value of Baseline Neutrophil-to-Lymphocyte Ratio in Advanced Breast Cancer Patients Treated with Trastuzumab Deruxtecan: A Real-World Study
Source: J Clin Med. 2026 Oct 29;15(21):8437. doi: 10.3390/jcm15218437 (PMC13764087; doi:10.3390/jcm15218437)

Supplementary Materials

Prognostic Value of Baseline Neutrophil-to-Lymphocyte Ratio in Advanced Breast Cancer Patients Treated with Trastuzumab Deruxtecan: A Real-World Study

Manuscript ID: jcm-4599780 · Analysis cohort: n = 92 · 39 progression-free survival events

This file contains Supplementary Tables S1–S3 and Supplementary Figures S1–S4, in the order listed in the Supplementary Materials section of the manuscript. Every title and legend below reproduces verbatim the wording listed in that section. Figure S3 is the NLR distribution and sensitivity-analysis figure and Figure S4 is the subgroup forest plot, consistent with the citations in Sections 3.9 and 3.10 of the manuscript.

Supplementary Table S1. Variable definitions with coding, reference categories and available sample sizes.

Variable	Definition and coding	Reference category	n available
Baseline NLR	Neutrophils ÷ lymphocytes ($\times 10^9$/L), count within 7 d before first T-DXd; closest value if multiple	continuous	92
PFS	T-DXd initiation to progression per RECIST 1.1 or death; months	—	92
Event	Progression = 1, censored = 0	censored	92
ECOG PS	0–3 at T-DXd initiation; dichotomised ≥ 1	0	92
HR status	ER or PR ≥ 1% nuclear staining = positive	negative	92
Visceral metastasis (primary, source-record field)	The composite visceral-metastasis variable as recorded in the source dataset (1 = visceral-positive, 0 = not); not re-derived from the site-specific liver/lung/brain variables. As documented in the Table 1 footnote, this field differs from the liver/lung/brain union in 18 of 92 patients (11 flagged visceral-positive with only nodal, chest-wall or cutaneous involvement; 7 flagged visceral-negative despite liver, lung or brain involvement).	absent	92
Visceral metastasis (reconstructed, sensitivity)	Liver, lung or brain involvement before first T-DXd (= 1 if any site positive); each site variable verified against the source metastatic-site text field (92/92 concordant). Reported as a sensitivity definition.	absent	92
Liver / lung / brain metastasis	Site-specific involvement before first T-DXd	absent	92
Metastatic sites	Count of involved sites; dichotomised ≥ 2 and ≥ 3	< 2 / < 3	92
BMI	Weight ÷ height², computed from height and weight recorded in the source record; dichotomised ≥ 25 kg/m²	< 25	84
Age	Years at T-DXd initiation; dichotomised > 60 (source-field threshold)	≤ 60	92
DFS	Curative-intent treatment to first recurrence; months; dichotomised ≥ 2 y	< 2 y	57
De novo stage IV	Metastatic presentation at initial diagnosis	no	92
Ki-67	Primary-tumour Ki-67 ≥ 20% = high	< 20%	83
Prior anti-HER2 lines	Lines containing any anti-HER2 agent; dichotomised > 2	≤ 2	73
Total treatment lines	Total prior systemic treatment lines; dichotomised > 2	≤ 2	84
Prior chemotherapy lines	Salvage lines for metastatic disease; dichotomised ≥ 2	< 2	91
PLR	Platelets ÷ lymphocytes; dichotomised > 144	≤ 144	92
Centre	Format of anonymised identifier (prefixed = Peking University Cancer Hospital, numeric = Beijing Friendship Hospital)	Peking University Cancer Hospital	92

For every binary covariate the value was re-derived from its source variable at the time of analysis; patients with a missing or undefined source value are excluded from the analyses containing that covariate (complete-case, per-covariate) rather than being

assigned to either category, and no imputation was performed. The "n available" column gives the evaluable denominator for each covariate. An earlier draft had instead used pre-computed binary indicator fields in which missing source values had been recoded into one of the two categories; this was identified during revision and all affected variables were re-derived and all affected estimates recomputed.

Supplementary Table S2. Proportional-hazards test results for all model terms.

Model / variable	Time transform	Test statistic	P value	Interpretation
Univariable model: continuous NLR	rank	0.1056	0.7452	Assumption satisfied
Univariable model: continuous NLR	Kaplan–Meier	0.1391	0.7092	Assumption satisfied
Univariable model: continuous NLR	identity	0.1858	0.6664	Assumption satisfied
Clinically driven multivariable model: NLR (per unit)	rank	0.0083	0.9272	Assumption satisfied
Clinically driven multivariable model: ECOG PS ≥ 1 vs 0	rank	0.6171	0.4321	Assumption satisfied
Clinically driven multivariable model: Visceral metastasis (source-record field) vs none	rank	0.0005	0.9825	Assumption satisfied
Clinically driven multivariable model: HR-positive vs negative	rank	0.1278	0.7208	Assumption satisfied
Clinically driven multivariable model: Age > 60 vs ≤ 60	rank	0.0090	0.9242	Assumption satisfied

Tests based on Schoenfeld residuals with the indicated time transforms. A P value ≥ 0.05 indicates no evidence of a time-varying effect, i.e. the proportional-hazards assumption is not violated. The clinically driven multivariable model comprised NLR, ECOG performance status, visceral metastasis (source-record field), hormone receptor status and age > 60 years (n = 92, 39 events, 7.8 events per variable). Disease-free survival was not entered into this model because it is evaluable in only 57 of 92 patients; the model retaining it is reported in the main text as a complete-case sensitivity analysis. For the median-split form of NLR the rank-transform test gave P = 0.064, also indicating no violation.

Supplementary Table S3. Restricted cubic spline coefficients and knot placements.

Knots (k)	Knot placement (NLR percentiles and values)	Term	Coefficient	Std. error	P
3	10th, 50th, 90th: 1.2002, 2.1565, 3.5851	s0 (linear)	+0.66068	0.37872	0.0811
		s1 (non-linear)	-0.06416	0.06660	0.3354
4	5th, 35th, 65th, 95th: 1.0206, 1.7484, 2.3671, 4.8190	s0 (linear)	+0.68387	0.34762	0.0492
		s1 (non-linear)	-0.00740	0.06719	0.9123
		s2 (non-linear)	-0.07867	0.16937	0.6423
5	5th, 27.5th, 50th, 72.5th, 95th: 1.0206, 1.5293, 2.1565, 2.6067, 4.8190	s0 (linear)	+0.66294	0.34414	0.0541
		s1 (non-linear)	+0.00133	0.06444	0.9836
		s2 (non-linear)	-0.01441	0.11315	0.8987
		s3 (non-linear)	-0.12346	0.32734	0.7061

Non-linearity tests (likelihood-ratio comparison of the spline model against the linear specification, penaliser 0.01 applied to both to stabilise the spline fit):

Knots (k)	LRT χ^2	df	P (non-linearity)	AIC linear	AIC spline	Preferred model
3	0.9457	1	0.3308	282.17	283.22	Linear
4	1.5748	2	0.4550	282.17	284.59	Linear
5	1.7368	3	0.6288	282.17	286.43	Linear
Quadratic term (single extra parameter)	3.2594	1	0.0710	282.17	—	Linear

In every specification the linear model was preferred on the Akaike information criterion and no evidence of non-linearity was detected; the linear (per-unit) hazard ratio reported in the main text is therefore an appropriate functional form. Knots were placed at percentiles of the observed NLR distribution in the analysis cohort (median 2.1565, interquartile range 1.5192–2.7227). Spline terms are the restricted cubic spline basis functions s0 (linear) and s1…s(k−2) (non-linear basis), constructed with the median knot as the reference. These values are unchanged by the covariate re-derivation described above, because baseline NLR was complete in all 92 patients and the spline models contain NLR as the only covariate.

Supplementary Figure S1. Kaplan–Meier curves for progression-free survival stratified at the median of each individual blood component: neutrophil count, lymphocyte count, platelet count, platelet-to-lymphocyte ratio and NLR. Only the lymphocyte count and NLR showed a significant median-split association (log-rank p = 0.011 and 0.0096, respectively).

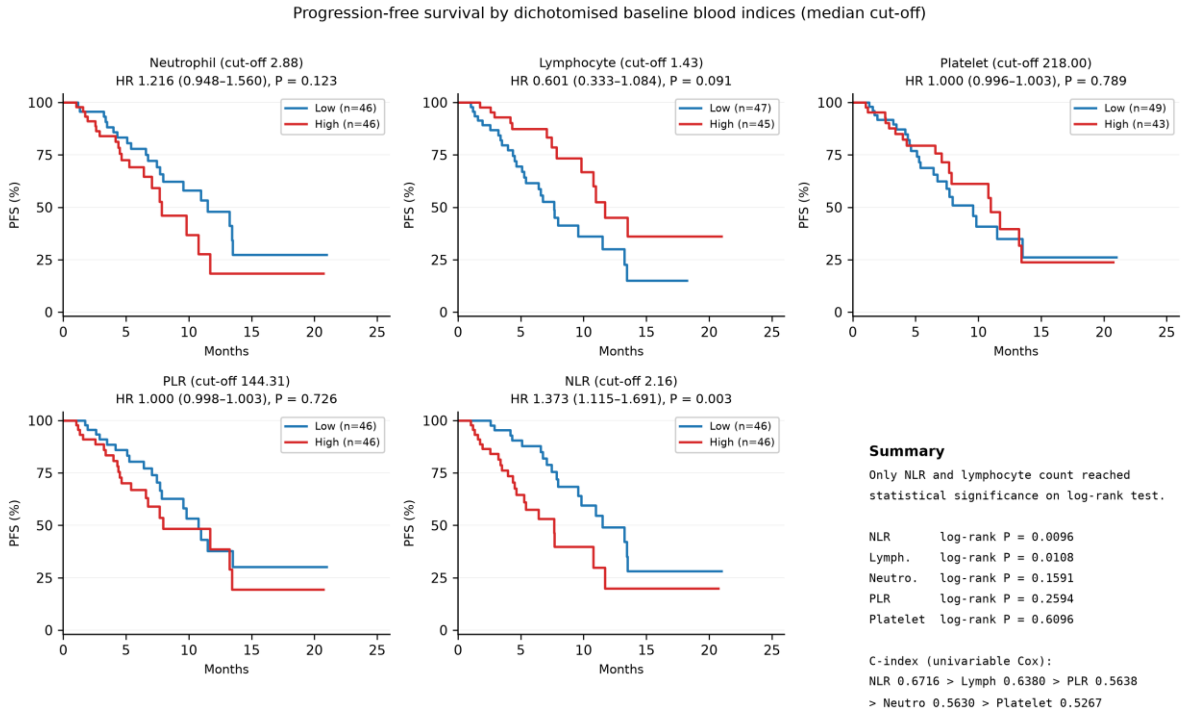

Supplementary Figure S2. Model fit (AIC) and discrimination (Harrell's C-index) across modelling strategies. NLR alone and NLR added to the clinical model outperform every single blood component and the clinical model without NLR.

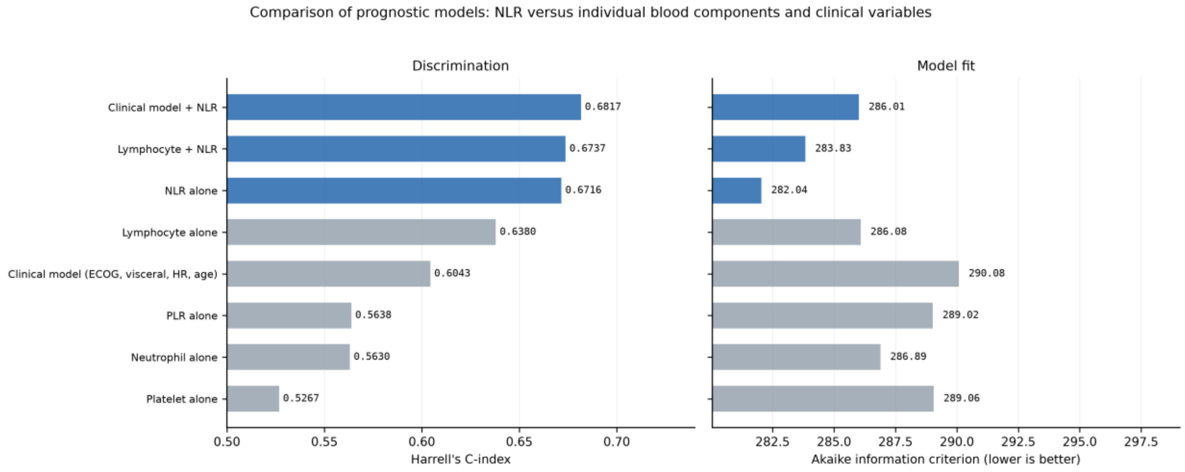

Supplementary Figure S3. (A) Distribution of baseline NLR with the median cut-off and interquartile and decile boundaries. (B) Sensitivity analyses of the primary endpoint: composite endpoint, alternative event definition, log(NLR), restriction to NLR ≤ 4, the relaxed timing window, and reinstatement of the single candidate with an unreliable baseline value on both the linear and log scales. The linear-scale estimate is sensitive to that single leverage point; the log-scale estimate is not.

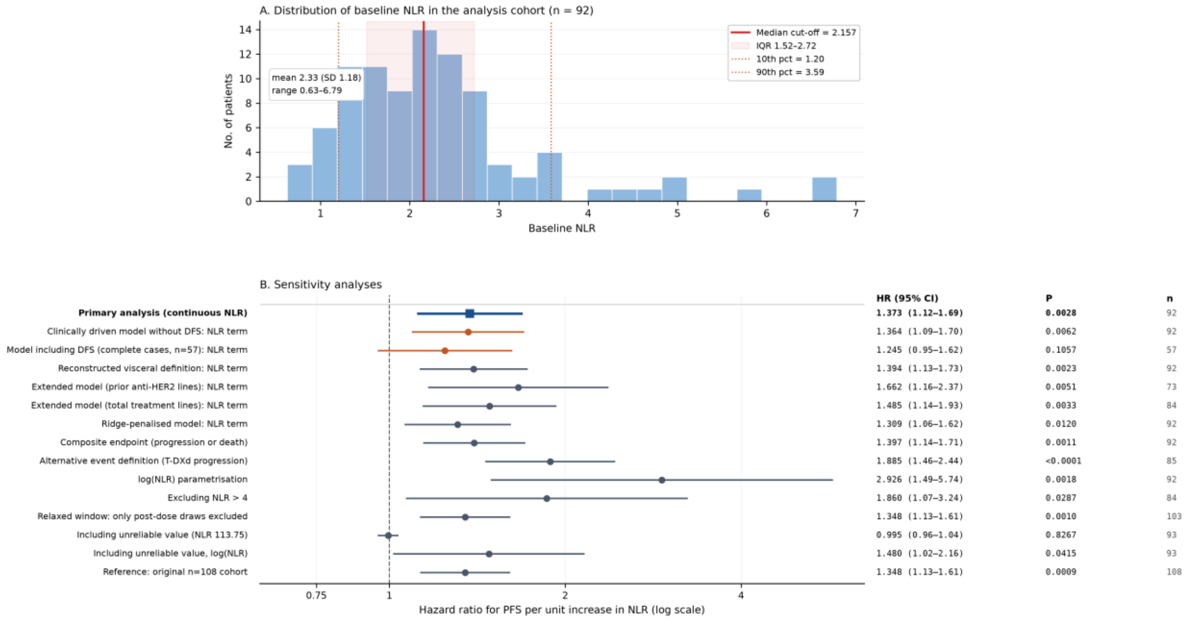

Supplementary Figure S4. Forest plot of exploratory subgroup analyses of the NLR–PFS association, with hazard ratios, 95% confidence intervals, subgroup p-values, interaction p-values and the number of patients and events per stratum. No interaction was significant before or after Benjamini–Hochberg correction across the seventeen tests (smallest raw p = 0.052; all q ≥ 0.372).

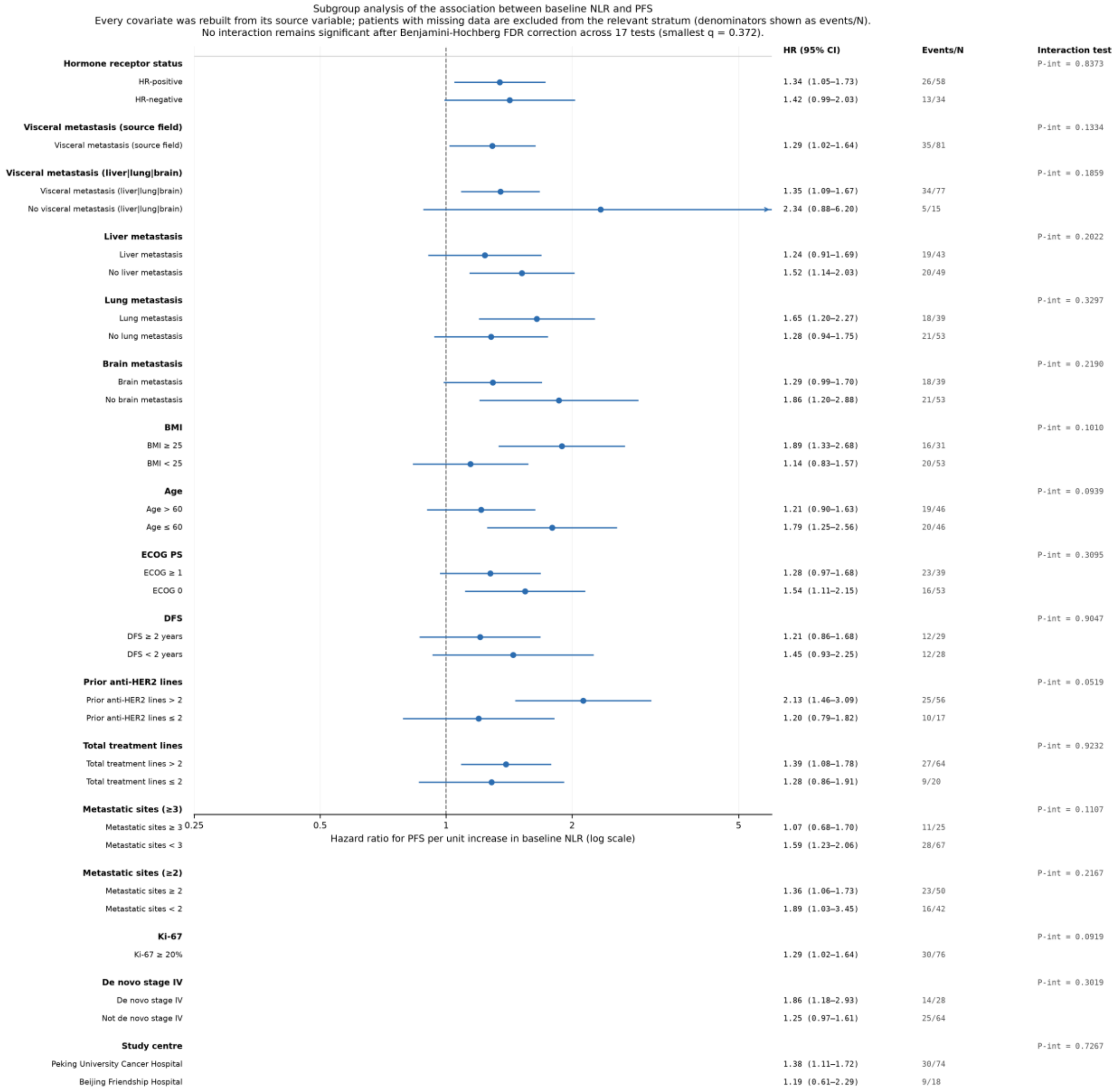

Supplement: Supplementary file 1 [file jcm-15-08437-s001.zip › jcm-4599780-supplementary.pdf]
